# Myosteatosis and sarcopenia are linked to autonomous cortisol secretion in patients with aldosterone-producing adenomas

**DOI:** 10.1038/s41440-024-01933-y

**Published:** 2024-10-14

**Authors:** Bo-Ching Lee, Yu-Ling Chang, Po-Ting Chen, Li-Wen Liu, Kao-Lang Liu, Chin-Chen Chang, Vin-Cent Wu, Yen-Hung Lin, On behalf of the TAIPAI Study Group, Vin-Cent Wu, Tai-Shuan Lai, Shih-Chieh Jeff Chueh, Shao-Yu Yang, Kao-Lang Liu, Chin-Chen Chang, Bo-Ching Lee, Shuo-Meng Wang, Kuo-How Huang, Po-Chih Lin, Yen-Hung Lin, Chi-Sheng Hung, Lian-Yu Lin, Shih-Cheng Liao, Ching-Chu Lu, Chieh-Kai Chan, Leay-Kiaw Er, Ya-Hui Hu, Che-Hsiung Wu, Yao-Chou Tsai, Zheng-Wei Chen, Chien-Ting Pan, Che-Wei Liao, Cheng-Hsuan Tsai, Yi-Yao Chang, Chen-Hsun Ho, Wei-Chieh Huang, Ying-Ying Chen

**Affiliations:** 1 Department of Medical Imaging, National Taiwan University Hospital and National Taiwan University College of Medicine, Taipei, Taiwan; 2 Department and Graduate Institute of Forensic Medicine, National Taiwan University College of Medicine, Taipei, Taiwan; 3 Department of Internal Medicine, National Taiwan University Hospital and National Taiwan University College of Medicine, Taipei, Taiwan; 4 National Taiwan University Hospital, Taipei, Taiwan; 5 Taipei Tzu-Chi Hospital, Buddhist Tzu Chi Medical Foundation, Taipei, Taiwan; 6 Department of Internal Medicine, National Taiwan University Hospital Yun-Lin Branch, Yun-Lin, Taiwan; 7 Department of Medicine, National Taiwan University Cancer Center, Taipei, Taiwan; 8 Department of Internal Medicine, National Taiwan University Hospital Jin-Shan Branch, New Taipei City, Taiwan; 9 Department of Cardiovascular Medicine, Far Eastern Memorial Hospital, New Taipei City, Taiwan; 10 Shin Kong Wu Ho-Su Memorial Hospital, Taipei, Taiwan; 11 Taipei Veterans General Hospital, Taipei, Taiwan; 12 MacKay Memorial Hospital, Taipei, Taiwan

**Keywords:** Myosteatosis, Sarcopenia, Body composition, Glucocorticoids, Autonomous cortisol secretion, Hyperaldosteronism

## Abstract

Patients with adrenal aldosterone-producing adenomas (APA) face elevated cardiovascular risks, especially when cortisol is co-secreted, yet the impact on muscle health remains unclear. Myosteatosis, characterized by fatty infiltration into muscles, is linked to cardiometabolic diseases and decreased survival. We aimed to investigate the association between autonomous cortisol secretion (ACS) in APA and muscle quantity and quality. In this study, we analyzed data from 228 APA patients undergoing laparoscopic adrenalectomy between 2009 and 2024, assessing muscle composition via computed tomography. Intermuscular adipose tissue (IMAT), skeletal muscle area and density, visceral and subcutaneous adipose tissue area at L3 were measured. Comparisons were made between ACS and non-ACS groups. We found that among 228 patients, 76 (33.3%) had ACS. Those with ACS exhibited significantly higher IMAT area (*P* = 0.042) and lower skeletal muscle area (*P* = 0.002) and density (*P* < 0.001). Multivariable regression confirmed ACS positively associated with IMAT area and negatively associated with skeletal muscle area and density. At 1-year follow-up, ACS patients (*n* = 15) experienced decreased IMAT area (*P* = 0.001) and increased skeletal muscle area (*P* = 0.031) post-adrenalectomy, while those without ACS (*n* = 29) showed no IMAT change but increased visceral (*P* < 0.001) and subcutaneous (*P* = 0.008) adipose tissue area. In summary, myosteatosis and sarcopenia are linked to ACS in APA patients, and these parameters improve following adrenalectomy.

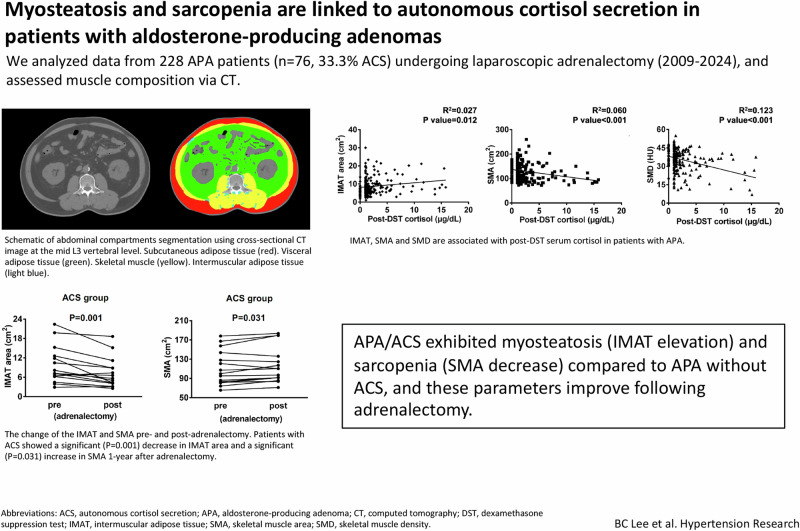

## Introduction

The prevalence of metabolic syndrome and obesity in patients with primary aldosteronism (PA) is high. Excess aldosterone results in cardiac remodeling and metabolic sequelae, including visceral obesity, insulin resistance, and impaired glucose homeostasis [1]. Concurrent autonomous cortisol secretion (ACS) is often underdiagnosed in PA due to incomplete screening. ACS not only leads to a higher prevalence of obesity and diabetes [2] but also independently increases cardiovascular events among patients with PA [3, 4]. Studies have indicated that in patients with ACS but without PA, increased cortisol secretion is linked to higher visceral adipose tissue (VAT) and reduced muscle quantity [5, 6]. However, little is known about ACS-induced changes in body composition among patients with PA. One study found no difference in total, visceral, and subcutaneous fat volumes between ACS and non-ACS groups in patient with PA [7]. Conversely, another study revealed that among patients with aldosterone-producing adenoma (APA), a subtype of PA, those with ACS exhibited significantly lower skeletal muscle area compared to those without ACS [8].

Myosteatosis, described as excess fat deposition in the skeletal muscle, is currently gaining attention because it provides a distinct perspective in sarcopenia research [9]. Multiple studies have demonstrated that myosteatosis is a negative prognostic factor in cancer and is associated with a higher complication rate in various diseases such as cardiovascular disease and hepatic steatosis [10]. While Cushing’s syndrome is widely recognized for its association with visceral obesity and muscle wasting, research on ACS cohorts is limited, with no prior investigations into the impact of PA [6, 11]. In addition, the relationship between myosteatosis and ACS has not been investigated. The aim of our study was to clarify the relationship between muscle fat content, especially myosteatosis, and ACS in patients with PA.

## Materials and methods

### Data sources

The multicenter Taiwan Primary Aldosteronism Investigators (TAIPAI) database maintains a standardized database of PA and prospectively collects biochemical, imaging, and clinicopathological data. The study population and criteria used for the diagnosis of PA were described in a previous publication [12]. The study protocol was approved by the Ethics Committee of the National Taiwan University Hospital. Informed consent was obtained from all participants prior to inclusion in the study.

### Study population

We conducted a retrospective analysis of 228 adult patients with APA who underwent unilateral adrenalectomy between January 2009 and April 2024. Both men and women aged >18 years were eligible for this study. Of these, 44 recruited patients underwent a follow-up abdominal CT-scan 1-year after post-adrenalectomy.

### Diagnosis criteria

1. Primary aldosteronism
  The diagnosis of PA was established in hypertensive patients by fulfillment of the following criteria : (1) autonomous excess aldosterone production evidenced by an aldosterone-renin ratio > 35 ng/mL/h; (2) plasma aldosterone concentration (PAC) > 16 ng/dL in a seated saline infusion test, or PAC/ plasma renin activity > 35 ng/dL shown in a captopril/losartan challenge test [12].
2. Aldosterone-producing adenoma
  The diagnosis of APA was based on (1) the evidence of PA at the screening tests mentioned above; (2) lateralization of aldosterone secretion with adrenal vein sampling confirmed; (3) histopathological evidence of adenoma after adrenalectomy; and (4) PA being corrected by adrenalectomy.
3. Patients with autonomous cortisol secretion
  Patients were categorized as having ACS based on an overnight 1 mg dexamethasone suppression test (DST). According to the guideline proposed by European Endocrinology Society, a cutoff point of 1.8 μg/dl is applied to exclude ACS [13]. ACS diagnosis required serum cortisol ≥5 μg/dL after a 1 mg DST. For levels between 1.8 and 4.9 μg/dL, at least one additional criteria such as ACTH < 10 pg/mL, nocturnal cortisol ≥5 μg/dL, low DHEA-S, or UFC > 70 μg/24-h were needed.
4. Post-adrenalectomy outcome

We used the Primary Aldosteronism Surgery Outcome (PASO) consensus criteria to classify the clinical and biochemical outcomes of adrenalectomy [14]. These criteria provide standardized definitions for complete, partial, and absent success based on blood pressure control, use of antihypertensive medications, and levels of plasma potassium, aldosterone, and renin. The outcomes were categorized accordingly as complete, partial, or absent success

### Biochemical measurements

Serum cortisol was measured using a chemiluminescent immunoassay (Architect, Abbott, VA, USA), and PAC and plasma renin activity were measured using commercial radioimmunoassay kits (Biochem Immunosystems, Bologna, Italy, and Stillwater, MN, USA, respectively). Anti-hypertensive medication and medication that may interfere with DST were discontinued prior to the study [12].

### Body composition analysis by computed tomography (CT)

**Fig. 1:**
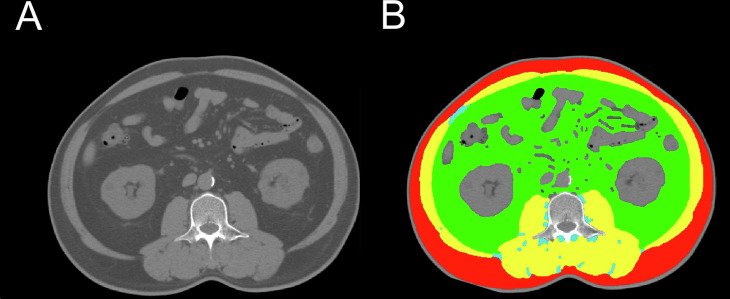
Schematic of abdominal compartments segmentation using semi-automatic software. The **A** cross-sectional computerized tomography image at the mid L3 vertebral level is quantified based on different densities of **B** subcutaneous adipose tissue (red), visceral adipose tissue (green), skeletal muscle (yellow), and intermuscular adipose tissue (light blue)

Various CT scanners were used for brain CT in this study, with detector ranges from 64 to 320. These scanners included LightSpeed VCT by GE Healthcare (Milwaukee, Wisconsin, USA), Brilliance 64 and Brilliance iCT 256 by Philips Healthcare (Best, The Netherlands), SOMATOM Sensation 64, SOMATOM Definition AS by Siemens Healthineers (Erlangen, Germany), and Aquilion ONE by Toshiba Medical Systems (Tokyo, Japan). All CT examinations were performed using the following parameters: 120 kVp and automated dose modulation. The imaging slice thickness was 5 mm, and the scan range was from the upper edge of the diaphragm to the lower pole of both kidneys. A blinded trained researcher with the guidance of two radiologists (B-CL and C-CC) analyzed the CT images using sliceOmatic image analysis software (version 5.0, Tomovision, Montreal, QC, Canada). For body composition analysis, using predefined Hounsfield units (HU) ranges, the total areas of skeletal muscle (SMA, −29 to 150 HU), visceral adipose tissue (VAT, −150 to −50 HU), subcutaneous adipose tissue (SAT, −190 to −30 HU), and intermuscular adipose tissue (IMAT, −190 to −30 HU) were measured at the mid L3 vertebral level of selected axial CT slices (Fig. 1); afterward, the SMA, VAT, SAT, and IMAT were manually tagged and quantified. Single-slice abdominal images at the L3 vertebra correlate strongly with whole-body volumes of skeletal muscles and adipose tissues, as reported in previous studies [15]. Skeletal muscle density (SMD) and IMAT density is defined by mean HU of the SMA and IMAT, respectively.

### Statistical analysis

All data are presented as the mean ± standard deviation unless otherwise specified. Baseline characteristics were compared using a two-sample *t*-test for continuous variables and a chi-square test for categorical variables. Multivariable analyses were performed using a multivariable linear regression model after adjustment for confounding factors. All parameters were systematically checked for collinearity. The linear relationship between two continuous variables was assessed using Pearson’s correlation. Comparisons of body composition parameters pre- and post-adrenalectomy were conducted using the Wilcoxon signed-rank test. All statistical analyses for significance and resulting *P* values were two-sided with *P* < 0.05. We utilized SPSS Statistics software version 25 (IBM, Armonk, NY, USA) for all analyses. GraphPad Prism 9 software was used for creating all figures.

## Results

Table 1 shows the demographic data and body composition parameters of each group of patients. The mean age of the individuals was 52.6 ± 10.6 years, and half (49.6%) of them were male. Of 228 patients with APA, 76 (33.3%) had ACS. APA patients with ACS were older (55.7 ± 10.4 vs. 51.0 ± 10.3, *P* = 0.002), with higher serum potassium (3.8 ± 0.6 mmol/L vs. 3.6 ± 0.5 mmol/L, *P* = 0.012), higher post-DST serum cortisol (5.2 ± 3.8 μg/dL vs. 0.9 ± 0.5, *P* < 0.001), less baseline adrenocorticotropic hormone (8.4 ± 7.2 pg/mL vs. 21.1 ± 12.2 pg/mL, *P* < 0.001), less dehydroepiandrosterone sulfate (2.7 ± 2.2 μmol/L vs. 4.2 ± 2.7 μmol/L, *P* < 0.001) compared to those without ACS. Other clinical characteristics, hypertensive medications, lipid profile and comorbidities related to metabolic and cardiovascular diseases showed no significant differences between both groups.

**Table 1:** Baseline characteristics and body composition parameters of APA patients with non-ACS and ACS status

Characteristics	Total (*n* = 228)	ACS group (*n* = 76)	Non-ACS group (*n* = 152)	*P* value
Age, years	52.6 ± 10.6	55.7 ± 10.4	51.0 ± 10.3	0.002**
Sex, Male (%)	113 (49.6%)	40 (52.6%)	73 (48.0%)	0.512
BMI, kg/m^2^	26.5 ± 4.9	25.8 ± 5.0	26.8 ± 4.9	0.117
Comorbidity				
Diabetes mellitus, *n* (%)	50 (21.9%)	22 (28.9%)	28 (18.4%)	0.070
Hyperlipidemia, *n* (%)	71 (31.1%)	26 (34.2%)	45 (29.6%)	0.479
Coronary artery disease, *n* (%)	17 (7.5%)	7 (9.2%)	10 (6.6%)	0.476
Duration of HTN, *y*	8.0 ± 7.3	8.9 ± 7.2	7.6 ± 7.3	0.244
Hypertension drugs, *n* (%)				
ACEI, *n* (%)	7 (3.1%)	3 (3.9%)	4 (2.6%)	0.587
ARB, *n* (%)	97 (42.5%)	30 (39.5%)	67 (44.1%)	0.507
Alpha-blocker, *n* (%)	45 (19.7%)	13 (17.1%)	32 (21.1%)	0.480
Beta-blocker, *n* (%)	59 (25.9%)	22 (28.9%)	37 (24.3%)	0.454
CCB, *n* (%)	146 (64.0%)	48 (63.2%)	98 (64.5%)	0.845
Vasodilator, *n* (%)	13 (5.7%)	4 (5.3%)	9 (5.9%)	0.840
Spironolactone, *n* (%)	74 (32.5%)	22 (28.9%)	52 (34.2%)	0.424
Diuretics, *n* (%)	16 (7.0%)	7 (9.2%)	9 (5.9%)	0.359
Clinical & Lab				
SBP, mm Hg	153.5 ± 20.0	152.9 ± 19.3	153.9 ± 20.4	0.740
DBP, mm Hg	92.6 ± 13.3	91.1 ± 12.9	93.4 ± 13.5	0.211
Fasting blood glucose, mg/dl	101.0 ± 20.3	101.1 ± 20.7	101.0 ± 20.1	0.975
Creatine, mg/dL	0.9 ± 0.3	0.9 ± 0.3	0.9 ± 0.3	0.800
Serum potassium level, mmol/L	3.6 ± 0.6	3.8 ± 0.6	3.6 ± 0.5	0.012*
Endocrine Profile				
Serum cortisol, μg/dL	9.8 ± 4.5	10.4 ± 4.7	9.5 ± 4.4	0.149
Post-DST serum cortisol, μg/dL	2.3 ± 3.0	5.2 ± 3.8	0.9 ± 0.5	<0.001***
Plasma aldosterone level, ng/dL	42.4 ± 30.4	47.2 ± 37.5	40.1 ± 26.0	0.143
Plasma renin activity, ng/mL/h	0.6 ± 0.9	0.6 ± 0.8	0.6 ± 0.9	0.712
Aldosterone–renin ratio	477.5 ± 1209.6	639.5 ± 1379.2	396.5 ± 1111.3	0.184
Baseline plasma ACTH, pg/mL	16.7 ± 12.3	8.4 ± 7.2	21.1 ± 12.2	<0.001***
DHEA-S, μmol/L	3.7 ± 2.7	2.7 ± 2.2	4.2 ± 2.7	<0.001***
24-h urinary free cortisol, μg	48.8 ± 52.1	60.8 ± 66.6	43.6 ± 43.7	0.099
Lipid profile				
Cholesterol, mg/dL	183.0 ± 35.4	182.9 ± 38.5	183.0 ± 33.8	0.975
Triglyceride, mg/dL	135.7 ± 142.0	146.1 ± 225.9	130.4 ± 66.9	0.436
HDL-C, mg/dL	48.5 ± 13.7	50.6 ± 13.8	47.6 ± 13.7	0.130
LDL-C, mg/dL	111.1 ± 27.8	111.9 ± 31.5	110.7 ± 25.9	0.776

Data are presented as the mean ± standard deviation

*ACEI* angiotensin-converting enzyme inhibitor, *ACS* autonomous cortisol secretion, *ACTH* Adrenocorticotropic hormone, *APA* aldosterone-producing adenoma, *ARB* angiotensin receptor blockers, *BMI* body mass index, *CCB* calcium channel blocker, *DBP* diastolic blood pressure, *DHEA-S* Dehydroepiandrosterone sulfate, *DST* dexamethasone suppression test, *HDL-C* high-density lipoprotein cholesterol, *HTN* hypertension, *LDL-C* low-density lipoprotein cholesterol, *SBP* systolic blood pressure

*P value < 0.05; **P value < 0.01; ***P value < 0.001

### Patients with APA/ACS showed myosteatosis and sarcopenia

We compared muscle quantity and quality between patients with ACS and those without ACS. The increased IMAT area reflects intermuscular fatty deposition, while decreased SMD indicates higher intramyocellular lipids (IMCL). In Table 2, the ACS group exhibited significantly higher IMAT area (9.6 ± 4.9 cm^2^ vs. 8.3 ± 4.4 cm^2^, *P* = 0.042), lower IMAT density (−29.3 ± 15.8 HU vs. −23.8 ± 13.5 HU, *P* = 0.006) and lower SMD (32.2 ± 10.6 HU vs. 37.3 ± 8.4 HU, *P* < 0.001) compared to non-ACS patients. Additionally, the ACS group showed significantly lower SMA (119.4 ± 39.1 cm^2^ vs. 135.3 ± 33.7 cm^2^, *P* = 0.002) compared to non-ACS group, indicating reduced muscle quantity. Other body composition parameters, including VAT area, VAT density, SAT area and SAT density, did not differ between the groups.

**Table 2:** Baseline characteristics and body composition parameters of APA patients with non-ACS and ACS status

Body composition	Total (*n* = 228)	ACS group (*n* = 76)	Non-ACS group (*n* = 152)	*P* value
**VAT area, cm**^**2**^	141.1 ± 86.8	144.3 ± 91.2	139.6 ± 84.8	0.702
**VAT density, HU**	−90.9 ± 12.6	−91.2 ± 13.6	−90.7 ± 12.1	0.808
**SAT area, cm**^**2**^	166.3 ± 76.3	165.0 ± 76.9	167.0 ± 76.2	0.855
**SAT density, HU**	−99.4 ± 8.4	−100.2 ± 10.2	−99.1 ± 7.3	0.400
**SMA, cm**^**2**^	130.0 ± 36.3	119.4 ± 39.1	135.3 ± 33.7	0.002**
**SMD, HU**	35.6 ± 9.5	32.2 ± 10.6	37.3 ± 8.4	<0.001***
**IMAT area, cm**^**2**^	8.7 ± 4.6	9.6 ± 4.9	8.3 ± 4.4	0.042*
**IMAT density, HU**	−25.6 ± 14.5	−29.3 ± 15.8	−23.8 ± 13.5	0.006**

Data are presented as the mean ± standard deviation

*ACS* autonomous cortisol secretion, *APA* aldosterone-producing adenoma, *HU* Hounsfield unit, *IMAT* intermuscular adipose tissue, *SAT* subcutaneous adipose tissue, *SMA* skeletal muscle area, *SMD* skeletal muscle density, *VAT* visceral adipose tissue

*P value < 0.05; **P value < 0.01; ***P value < 0.001

### ACS was independently associated with IMAT area, SMD and SMA

**Fig. 2:**
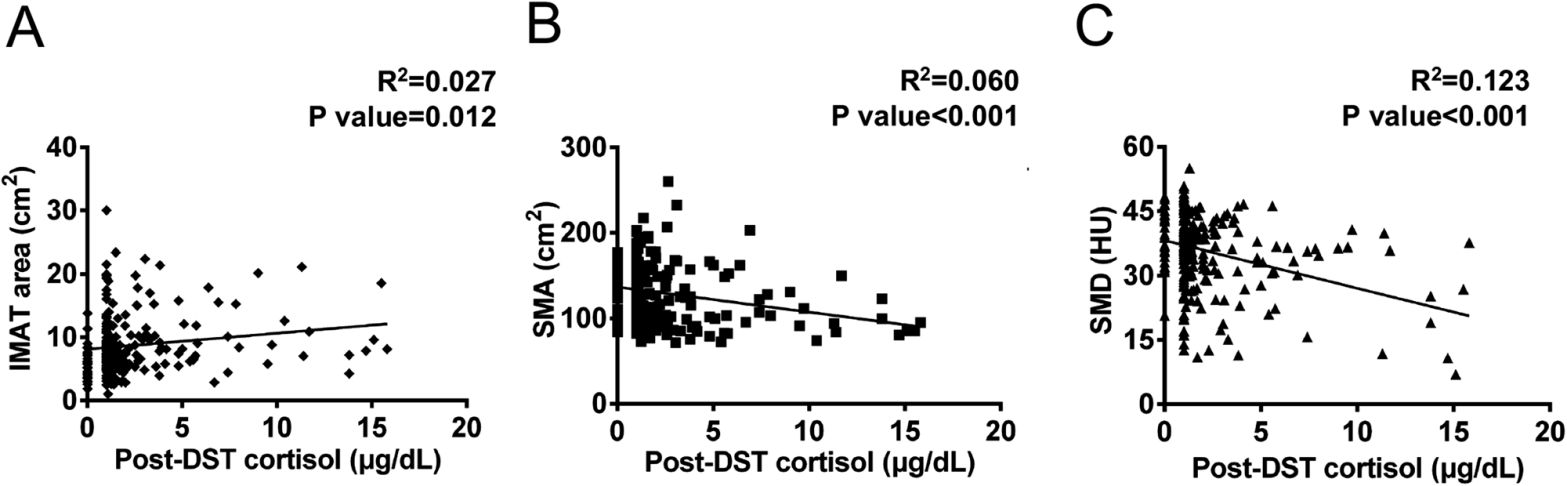
Associations of the body composition parameters with post-DST serum cortisol levels in patients with APA. The scatter plots illustrate the relationships between IMAT **A**, SMA **B** and SMD **C** and post-DST serum cortisol levels. APA aldosterone-producing adenoma, DST dexamethasone suppression test, IMAT intermuscular adipose tissue, SMA skeletal muscle area, SMD skeletal muscle density

We further examined the correlation between IMAT area, SMD, and SMA with post-DST serum cortisol levels and ACS status. In Fig. 2, post-DST serum cortisol levels positively correlated with IMAT area (R^2^ = 0.027, *P* = 0.012) and negatively correlated with SMA (R^2^ = 0.060, *P* < 0.001) and SMD (R^2^ = 0.123, *P* < 0.001). We then assessed if the association between muscle fat content, muscle quantity, and ACS was independent of metabolic factors. Bivariate analyses showed a significant association of ACS with SMA, SMD, IMAT area and IMAT density. After adjusting for confounders (age, sex, BMI, systolic BP, diabetes status, triglycerides, and cholesterol levels) in multivariate analyses, the relationship between ACS and SMA, SMD, IMAT area and IMAT density remained significant (all *P* < 0.05) (Table 3).

**Table 3:** Multivariable regression analyses of body composition parameters associated with the ACS status in patients with APA

	*Β*	β	95% CI, lower bound	95% CI, upper bound	*P* value
**SMA, cm**^**2**^					
Unadjusted	−15.918	−0.207	−25.774	−6.062	0.002**
Age, sex adjusted	−13.633	−0.177	−22.943	−4.322	0.004**
Multivariate model 1^a^	−8.919	−0.116	−16.082	−1.757	0.015*
Multivariate model 2^b^	−9.187	−0.120	−16.328	−2.045	0.012*
**SMD, HU**					
Unadjusted	−5.117	−0.255	−7.661	−2.572	<0.001***
Age, sex adjusted	−3.525	−0.176	−5.869	−1.181	0.003**
Multivariate model 1^a^	−3.681	−0.183	−5.961	−1.401	0.002**
Multivariate model 2^b^	−3.738	−0.186	−6.054	−1.422	0.002**
**IMAT area, cm**^**2**^					
Unadjusted	1.312	0.134	0.045	2.579	0.042*
Age, sex adjusted	1.308	0.134	0.008	2.608	0.049*
Multivariate model 1^a^	1.544	0.158	0.243	2.845	0.020*
Multivariate model 2^b^	1.605	0.151	0.163	2.937	0.018*
**IMAT density, HU**					
Unadjusted	−5.560	−0.181	−9.509	−1.610	0.006**
Age, sex adjusted	−4.318	−0.141	−8.212	−0.423	0.030*
Multivariate model 1^a^	−4.464	−0.146	−8.370	−0.559	0.025*
Multivariate model 2^b^	−4.638	−0.151	−8.574	−0.702	0.021*

*ACS* autonomous cortisol secretion, *APA* aldosterone-producing adenoma, *B* unstandardized regression coefficient, *β* standardized regression coefficient, *HU* Hounsfield unit, *IMAT* intermuscular adipose tissue, *SMA* skeletal muscle area, *SMD* skeletal muscle density

^a^Multivariate model 1 was adjusted for age, sex, systolic blood pressure, body mass index, and diabetes

^b^Multivariate model 2 was adjusted for triglyceride and cholesterol levels in addition to the factors included in Model 1

*P value < 0.05; **P value < 0.01; ***P value < 0.001

### Improvement of IMAT area and SMA in patients with APA/ACS post-adrenalectomy

A total of 44 patients underwent a follow-up abdominal CT scan one year after adrenalectomy. The rate of complete or partial clinical success is 73.3% (11/15) for the ACS group and 93.1% (27/29) for the non-ACS group; while the rate of complete or partial biochemical success is 80.0% (12/15) for the ACS group and 86.2% (25/29) for the non-ACS group, all without significant difference. Table 4 and Supplemental Fig. 1 illustrate that the ACS group (*n* = 15) exhibited a significant decrease in IMAT area (9.7 ± 5.7 cm^2^ to 7.3 ± 4.7 cm^2^, *P* = 0.001) and an increase in SMA (111.4 ± 36.1 cm^2^ to 117.0 ± 37.1 cm^2^, *P* = 0.031), with no change in IMAT density, SMD, VAT area, VAT density, SAT area and SAT density. Conversely, patients without ACS (*n* = 29) showed no alterations in IMAT area, IMAT density, VAT density, SAT density, SMA, or SMD but had significant increases in VAT area (132.9 ± 67.8 cm^2^ to 169.4 ± 82.6 cm^2^, *P* < 0.001) and SAT area (165.6 ± 67.4 cm^2^ to 191.5 ± 99.3 cm^2^, *P* = 0.008).

**Table 4:** The change of body composition parameters pre- and post-adrenalectomy in patient with APA

	Pre-adrenalectomy	Post-adrenalectomy	*P* value
**ACS group (*n*** = **15)**			
VAT area, cm^2^	154.4 ± 95.1	168.2 ± 108.4	0.233
VAT density, HU	−91.2 ± 11.7	−94.0 ± 10.7	0.053
SAT area, cm^2^	132.4 ± 51.1	141.3 ± 58.2	0.293
SAT density, HU	−99.2 ± 7.3	−102.5 ± 8.1	0.173
SMA, cm^2^	111.4 ± 36.1	117.0 ± 37.1	0.031*
SMD, HU	33.4 ± 13.2	32.2 ± 10.8	0.191
IMAT area, cm^2^	9.7 ± 5.7	7.3 ± 4.7	0.001**
IMAT density, HU	−28.2 ± 17.7	−30.7 ± 15.7	0.650
**Non-ACS group (*n*** = **29)**			
VAT area, cm^2^	132.9 ± 67.8	169.4 ± 82.6	<0.001***
VAT density, HU	−92.2 ± 11.9	−93.2 ± 10.8	0.405
SAT area, cm^2^	165.6 ± 67.4	191.5 ± 99.3	0.008**
SAT density, HU	−101.1 ± 7.1	−102.2 ± 3.6	0.611
SMA, cm^2^	128.2 ± 30.4	130.1 ± 32.8	0.495
SMD, HU	36.5 ± 6.5	36.9 ± 7.4	0.581
IMAT area, cm^2^	8.6 ± 5.1	9.0 ± 6.0	0.275
IMAT density, HU	−25.9 ± 13.6	−28.5 ± 13.0	0.074

Data are presented as the mean ± standard deviation

*ACS* autonomous cortisol secretion, *APA* aldosterone-producing adenoma, *HU* Hounsfield unit, *IMAT* intermuscular adipose tissue, *SAT* subcutaneous adipose tissue, *SMA* skeletal muscle area, *SMD* skeletal muscle density, *VAT* visceral adipose tissue

*P value < 0.05; **P value < 0.01; ***P value < 0.001

## Discussion

### Myosteatosis and sarcopenia is associated with ACS

Disruption of muscular metabolism is associated with metabolic impairment in hypercortisolism. Nevertheless, research on myosteatosis in ACS has not been conducted to date [16]. In this study, we used the IMAT area and SMD as indicators for evaluating myosteatosis. We demonstrated a previously unrecognized association between ACS and myosteatosis in APA patients, distinct from sarcopenia, providing independent prognostic information in various diseases [10]. Furthermore, this association remained significant after adjusting for multiple metabolic confounders. Importantly, the detrimental change in body composition parameter showed improvement after adrenalectomy, highlighting the efficacy of targeted therapy for APA patients. On the other hand, we found that visceral fat area increased after adrenalectomy in non-ACS patients with APA, which seems consistent with the findings of a prior study [17].

Although most studies only used SMD to diagnose myosteatosis [10], IMAT and SMD could have different physiological implications and clinical relevance in patients. They represent different fat content and distribution patterns. IMAT is an extramyocellular lipid deposition within muscles’ fascia and can be directly measured via CT segmentation [10]. IMCL refers to microscopic fat infiltration within myocytes, reflected by lower SMD on CT scans. This is because muscle radiodensity decreases as intramuscular fat deposition increases [18]. Our result showed that IMAT and IMCL mostly have the same tendency but are not always congruent, such as among patient after adrenalectomy, which suggests that different physiological mechanisms may cause the accumulation or resolution of IMAT and IMCL. The precise mechanisms of glucocorticoid-mediated IMAT and IMCL deposition have not been fully established. IMAT is responsible for mediating insulin resistance [19] and is involved in proinflammatory pathways in muscle, and further studies are needed to explore its prognostic implication in PA patients.

Moreover, we observed that the ACS group exhibited lower skeletal muscle quantity compared to the non-ACS groups among patients with APA. This aligns with findings from a prior study comparing ACS status in APA patients, which similarly reported significantly lower skeletal muscle area in those with ACS [8]. Interestingly, other studies involving general PA patients or nonfunctioning adrenal incidentaloma did not demonstrate such an association between muscle mass and ACS status [7, 20].

### Post-adrenalectomy changes of body composition

Another notable aspect of our study was the post-adrenalectomy improvement in IMAT and SMA. We observed a decrease in IMAT and an increase in SMA among the ACS group, contrasting with the non-ACS group where no significant changes were noted. This trend was not observed in SMD. Previous research has indicated that metabolic factors such as hypertension, BMI, and metabolic syndrome improve in ACS patients post-adrenalectomy, while cardiometabolic parameters remain unchanged or worsen in nonfunctioning adrenal incidentaloma patients [4]. Our findings suggest that IMAT may hold more clinical relevance than SMD after adrenalectomy in ACS patients. However, given the limited sample size, further validation in larger cohorts is warranted.

Additionally, we observed an increase in VAT and SAT after adrenalectomy in the non-ACS group, consistent with findings from other studies. One study reported significant weight gain in APA patient post-adrenalectomy [21], while another noted increased abdominal adipose tissue after adrenalectomy, particularly in those with KCNJ5 mutations [22]. This may be due to aldosterone-induced adipose tissue shrinkage and fibrosis in APA patients, as indicated by elevated fibronectin and collagen I protein levels [23, 24]. Moreover, APA patients experience increased sympathetic activation, which could lead to higher energy expenditure and reduced adipose tissue size [25]. Post-adrenalectomy, these effects are reversed, resulting in an increase in SAT and VAT.

### Visceral fat is not associated with ACS

In theory, glucocorticoids bind to adipocyte glucocorticoid receptors and induce adipogenesis, especially in the VAT. Intriguingly, our results showed no significant difference in VAT between the ACS and non-ACS groups, consistent with prior studies [8, 11, 20]. There are several possible explanations for this. First, glucocorticoid signaling is polymorphic and differs among people. Hence, using an absolute cut-off value of serum cortisol level to diagnose ACS may not suit everyone because the same cortisol level did not reflect the same effect. Second, the effect of aldosterone on VAT should also be considered. Recent research indicates lower VAT in APA patients compared to those with essential hypertension, suggesting a potential counterbalancing effect between cortisol and aldosterone in APA/ACS [17]. These data further support that both cortisol and aldosterone play complex roles in adipogenesis.

### Limitations

This retrospective study had several limitations. Firstly, the limited number of participants with follow-up abdominal CT scans reduced statistical power. Secondly, ACS can be clinically latent, making it challenging to ascertain the duration of cortisol excess prior to imaging, which may impact body composition redistribution. Additionally, the lack of standardized cutoff values and complementary diagnostic criteria in ACS protocols may introduce bias [12]. Thirdly, there is no standardized diagnostic consensus for myosteatosis. While most studies utilize abdominal CT and analyze body composition at the L3 level due to its correlation with whole-body muscle mass, we adhered to this methodology.

## Conclusions

We found that high muscle fat content and low muscle mass were independently associated with APA/ACS after adjusting for metabolic factors. These adverse changes improved following adrenalectomy, underscoring the efficacy of targeted therapy for PA patients. Given the accessibility of routine abdominal CT scans for APA patients, utilizing IMAT area and SMD as myosteatosis indicators to predict cardiovascular risk in ACS/APA could be beneficial. Assessing muscle quality at baseline and longitudinally may offer valuable insights. Further research, including ‘muscular fat mapping,’ is needed to elucidate skeletal muscle quality’s role in preventing cardiovascular and metabolic sequelae.

## Supplementary information

Supplemental Figure 1

Supplemental Figure 1
