# Supplementary material for: Myosteatosis and sarcopenia are linked to autonomous cortisol secretion in patients with aldosterone-producing adenomas
Source: Hypertens Res. 2024 Oct 14;48(2):519–28. doi: 10.1038/s41440-024-01933-y (PMC11794128; doi:10.1038/s41440-024-01933-y)
Supplement: Supplementary file 1 — Supplemental Figure 1 [file 41440_2024_1933_MOESM1_ESM.docx]

**Supplemental Figure 1.** The change of the IMAT area and SMA pre- and post-adrenalectomy. (A) Patients with ACS showed a significant (P=0.001) decrease in IMAT area 1-year after adrenalectomy. (B) Patients with ACS showed a significant (P=0.031) increase in SMA 1-year after adrenalectomy. (C, D) Patients without ACS showed similar IMAT area and SMA 1-year after adrenalectomy. ACS, autonomous cortisol secretion; IMAT, intermuscular adipose tissue; SMA, skeletal muscle area.
