# Supplementary figures and images for: Myosteatosis and sarcopenia are linked to autonomous cortisol secretion in patients with aldosterone-producing adenomas
Source: Hypertens Res. 2024 Oct 14;48(2):519–28. doi: 10.1038/s41440-024-01933-y (PMC11794128; doi:10.1038/s41440-024-01933-y)

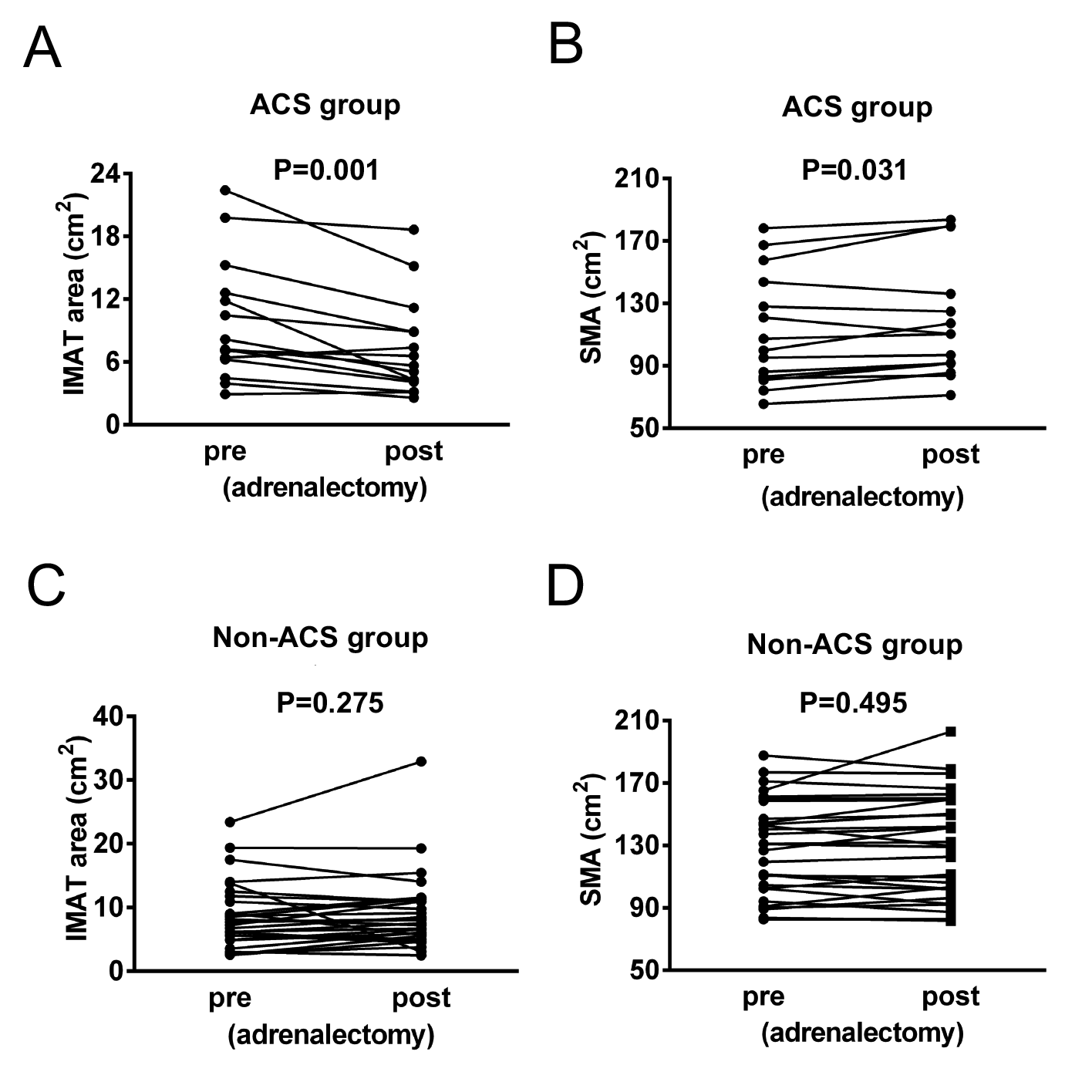

Supplement: Supplementary file 2 — Supplemental Figure 1 [file 41440_2024_1933_MOESM2_ESM.tif]
